# The hidden influence of large particles on ocean colour

**DOI:** 10.1038/s41598-021-83610-5

**Published:** 2021-02-17

**Authors:** Emlyn J. Davies, Sünnje L. Basedow, David McKee

**Affiliations:** 1 SINTEF Ocean, Trondheim, Norway; 2 The Arctic University of Norway, Tromsø, Norway; 3 University of Strathclyde, Glasgow, UK

**Keywords:** Ocean sciences, Optics and photonics

## Abstract

Optical constituents in the ocean are often categorized as water, phytoplankton, sediments and dissolved matter. However, the optical properties of seawater are influenced, to some degree, by scattering and absorption by *all* particles in the water column. Here we assess the relevant size ranges for determining the optical properties of the ocean. We present a theoretical basis supporting the hypothesis that millimetre-size particles, including zooplankton and fish eggs, can provide a significant contribution to bulk absorption and scattering of seawater and therefore ocean color. Further, we demonstrate that existing in situ instruments are not capable of correctly resolving the impact of such large particles, possibly leading to their optical significance being overlooked. These findings refresh our perspective on the potential of ocean color and invite new applications of remote sensing for monitoring life close to the ocean surface.

## Introduction

**Figure 1:**
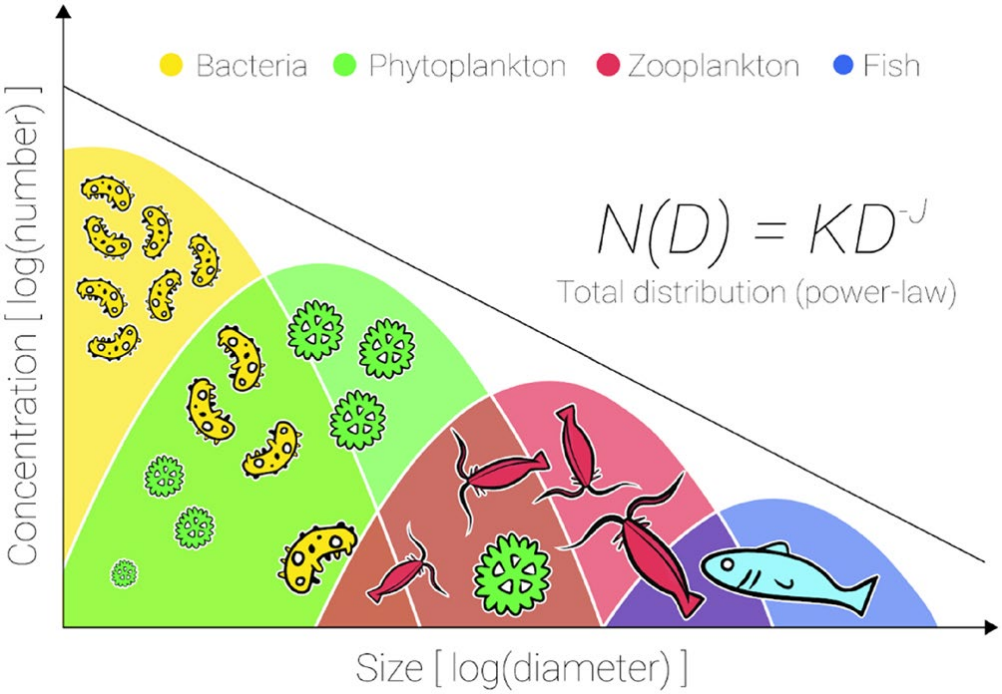
Natural particle size distributions in the ocean broadly follow a power law over many orders of magnitude, from viruses and bacteria to fish and whales. Non-living material contained in the PSD may also include marine snow, detritus, sediment and microplastic. The power law PSD is the sum of log-normal distributions for each sub-population, four examples of which are illustrated in this figure. N is the number of particles of diameter, D; K is the number of 1 µm particles per volume; J is the slope of the power-law distribution.

The oceans are home to a vast array of organisms with sizes spanning many orders of magnitude^1^. Particle size distributions (PSDs), graphically represented in Fig. 1, broadly follow a power law covering everything from viruses to whales, with abundances decreasing logarithmically with size^2–5^. Each particle sub-population typically follows a log-normal distribution, with the sum forming the overall power law and with scope for deviations from power law when a particular sub-population becomes unusually dominant e.g. in the case of an algal bloom or a zooplankton swarm^3^. Changes in sub-population concentrations affect the power-law exponent, *J*, of the total size distribution and alter the relative contributions of large and small particles to the total absorption, scattering or backscattering. Optically clear oceanic waters will generally present lower particle concentrations than productive or turbid coastal waters, and increased abundance of large flocculated material closer to the coast often causes the slope of the PSD to become shallower than offshore phytoplankton-dominated waters^6–11^.

Ocean color remote sensing from satellites is now routinely used for measurements of global-scale primary production^12–14^. Climate-driven changes in phytoplankton photosynthesis can be estimated from time-series of such estimates^15,16^. Determination of the content of the near-surface ocean based on its color comes, unsurprisingly, with some substantial assumptions and simplifications regarding interactions between particles and light, often leading to a debate over the accuracy of the vital global climate processes that are inferred^17^. Fully understanding the various contributions to ocean color signals is crucial for developing robust algorithms required for climate and ecosystem applications, and calibration/validation of new remote sensing techniques^18,19^.

**Figure 2:**
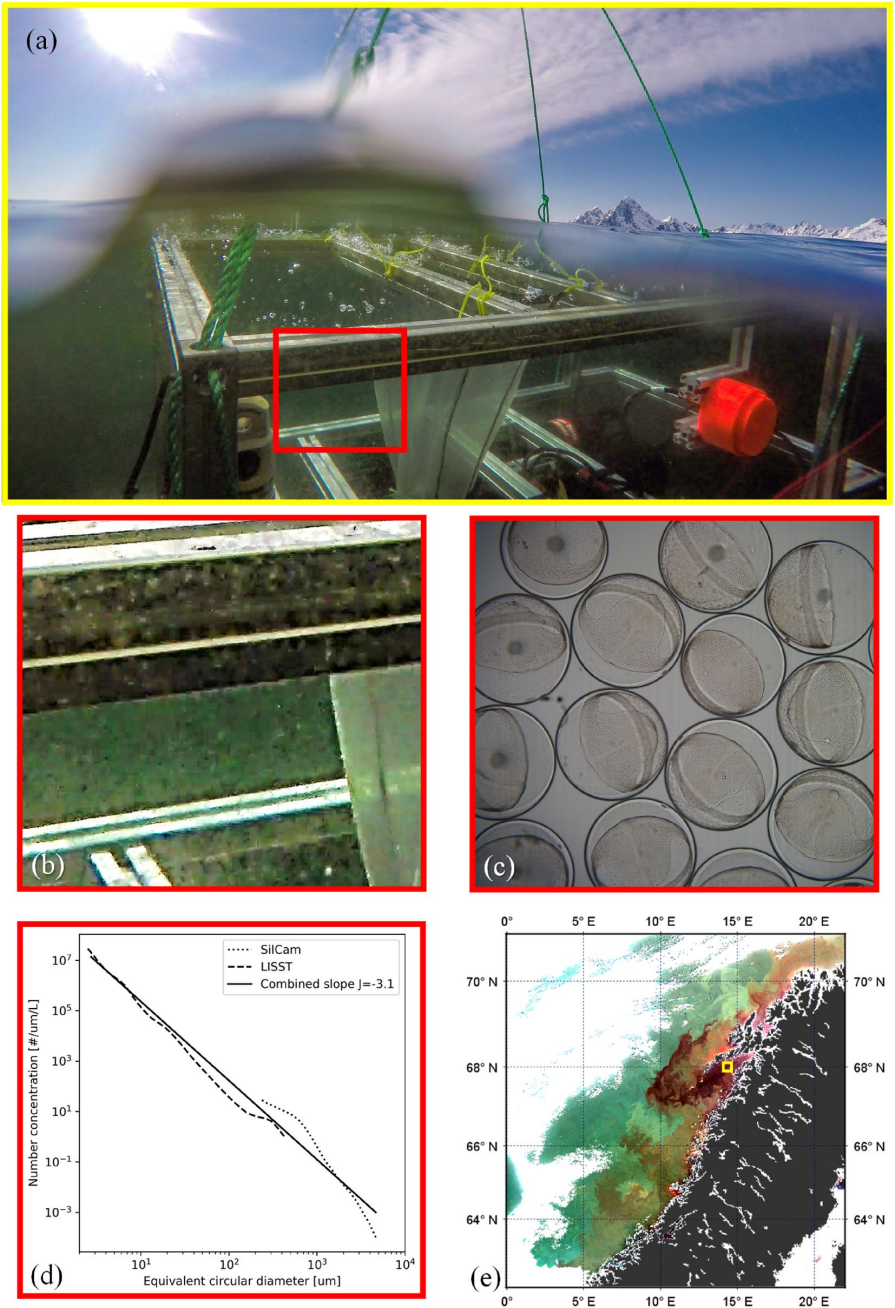
(**a**) A still image from a GoPro video captures a wave breaking over an instrument cage deployed off the Norwegian coast in April 2019. The image shows very high abundances of large particles, in this case primarily fish eggs and copepods, absorbing and scattering light. (**b**) Zooming in to an enhanced underwater region of the photo highlights the high abundance of large particles. (**c**) Fish eggs like these are visually coloured a light brown, preferentially absorbing blue light and are typically ~ 1.2 mm in diameter. (**d**) Analysis of images from an in situ Silhouette Camera^31^ and LISST-100x^32^ mounted on the frame allows enumeration of high abundances of large particles, showing the average size distribution in the upper 10 m below the sea surface. (**e**) An enhanced RGB image from the MODIS satellite from 5th April 2019 reveals strongly coloured waters in this region during the same period (data: NASA GSFC, processed by McKee using SeaDAS v7, cruise support from NEODAAS (PML) gratefully acknowledged).

The optical properties of the ocean are traditionally considered to be determined by the effect of dissolved organic materials and numerically abundant phytoplankton, detritus and other sediment and colloidal particles^2,20,21^. However, recent evidence suggests that larger but less abundant particles such as mm-sized particles (including zooplankton) may also be present in high concentrations near the surface^22^, so much so that they can influence ocean colour signals^23–27^. However, a discrepancy between remote sensing signals and in situ inherent optical property (IOP) measurements remains^23^. It appeared that remote sensing signals could have be influenced by large particle absorption while IOP measurements may not have been sensitive to this class of particles. In the field, one can visually observe large particles suspended in seawater with the naked eye, especially in coastal environments, and it is possible to detect their presence with simple cameras (Fig. 2). There are also several recorded and anecdotal observations of relatively large zooplankton, such as *Calanus* spp. and krill species, causing seawater to visually turn “a conspicuous reddish hue”^28^ or “yellowish tint”^29^. This raises the intriguing possibility that the ocean optics community has overlooked large particles as an important set of contributors to ocean colour signals.

Here we address two fundamentally important questions: (1) Is there a sound theoretical basis to support the suggestion that large (mm-class), but relatively sparse, coloured particles can significantly influence bulk IOPs of seawater? (2) If large particles are theoretically capable of influencing IOPs, why might in situ sensors fail to resolve their impact? The first issue can be investigated using Mie modelling^2^ following the approach of Davies et al*.*^30^. There it was demonstrated that scattering and backscattering signals can be influenced by a broad range of particle sizes (0.02–2000 µm). However, the effect of varying the imaginary refractive index, *n*_*i*_, on absorption was only very briefly examined and was shown to reduce the largest significant size for backscattering to ~ 150 µm for *n*_*i*_ = 0.001*i*. This effect requires further elucidation, and the general sensitivity of the absorption coefficient to particle size has not been systematically explored in this manner. The second question is more difficult to fully resolve, but we show that small sample volumes of IOP sensors are potentially a limiting factor in capturing the effect of large colored particles on bulk IOPs.

## Results

### Do large particles influence bulk optical properties?

**Figure 3:**
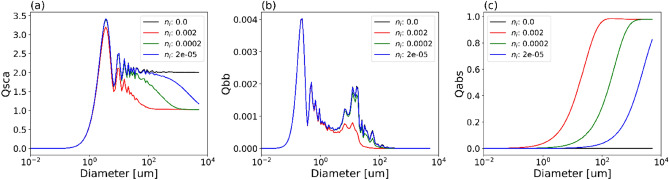
Increasing the imaginary refractive index (*n*_*i*_) reduces efficiency factors for (**a**) scattering, and (**b**) backscattering for particle sizes greater than 1–10 μm. (**c**) Increasing *n*_*i*_ results in particles achieving saturation efficiency (close to an absorption efficiency of 1) at smaller diameters.

We examined the effect of varying particle size and imaginary refractive index, *n*_*i*_, on efficiency factors for scattering, backscattering and absorption (Fig. 3). This was modelled using Mie theory, and expands on previous sensitivity studies^2,30^ to account for the full range of particle sizes and PSD slopes—we discuss the limitations of this approach within the Methods section. It appeared that large particles can be highly efficient absorbers, even if they are only lightly colored (low *n*_*i*_). Particle scattering efficiency for large particles converges towards 2 when *n*_*i*_ is zero (non-absorbing) but drops by almost 50% when *n*_*i*_ increases (Fig. 3a). The impact of *n*_*i*_ is less significant for backscattering (Fig. 3b), with only small decreases observed for particles in the range 1–100 μm and is only significant for the highest value of *n*_*i*_ used. In contrast, absorption efficiency is highly dependent on *n*_*i*_, with large particles (> 100 μm) reaching saturation efficiency close to 1 when *n*_*i*_ increases. In all cases it is the efficiencies of the largest particles that exhibit the most sensitive responses to changes in the imaginary refractive index.

**Figure 4:**
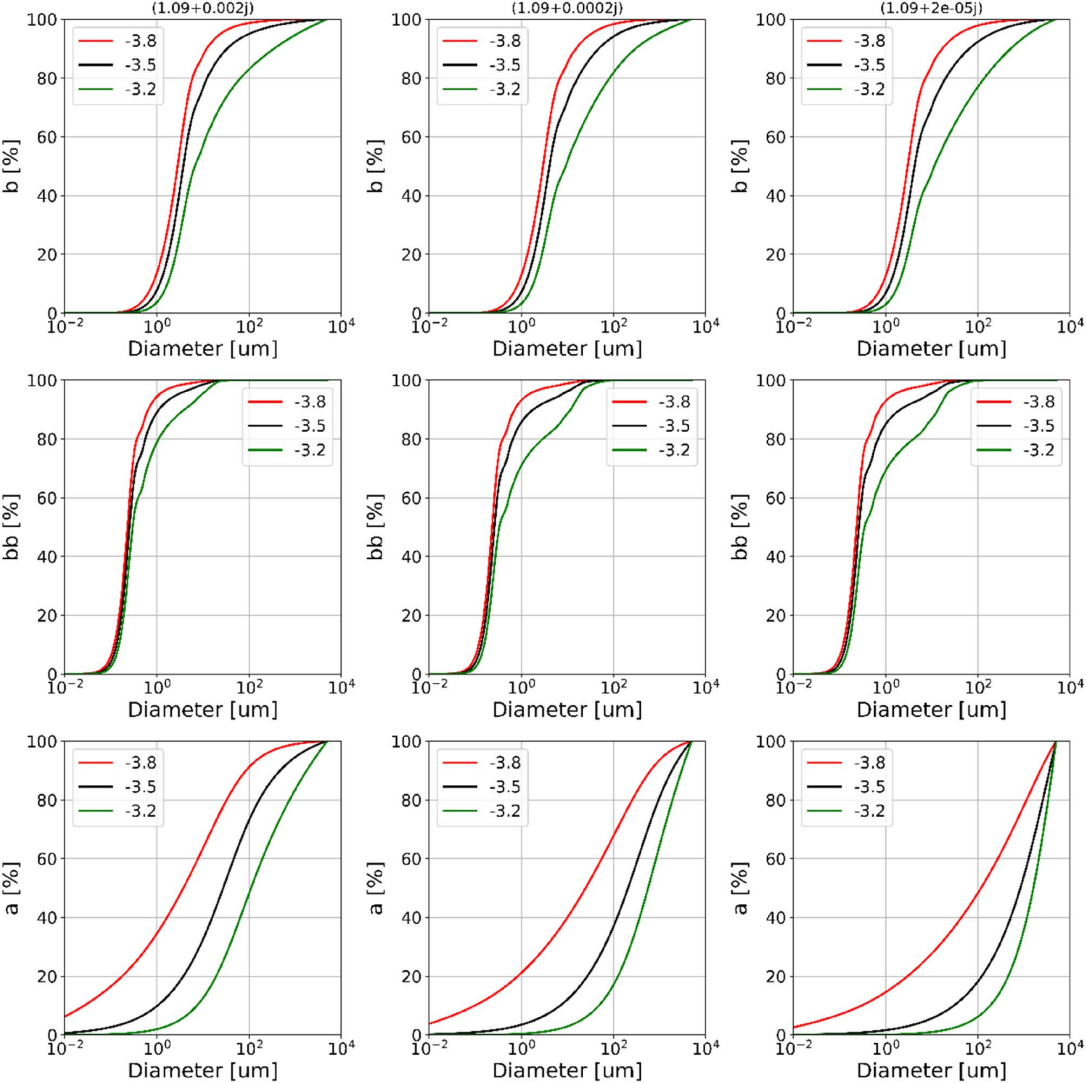
Changing imaginary refractive index (columns) on cumulative contributions to (top row) scattering, (middle row) backscattering, and (bottom row) absorption. Red, black and green lines represent PSD slopes of 3.8, 3.5 and 3.2 respectively. Failure to reach a horizontal asymptote implies that the range of significant particle sizes extends beyond the range of sizes simulated here (0.001–5000 µm).

For the sake of simplicity, we model PSDs using the power law function: $N\left(D\right)=K{D}^{-J}$ (introduced with Fig. 1). Natural particle PSDs have been shown to have power law slopes varying by as much as 2.64–6.68^33^, but values for coastal waters typically fall within a more restricted range of 3.2–3.8^34^. We used this more conservative range of power law slopes along with a range of realistic *n*_*i*_ values to establish the potential impact of imaginary refractive index on cumulative contributions to bulk IOPs (Fig. 4). Note that this conservative approach represents a reduced range of variability in PSD slope. We also operate under the assumption that refractive indices are consistent across the entire particle population and that the PSD is well represented by a power law, both of which are major simplifications on the complexity found in nature. More complex models should be considered for further study^35,36^, however, we deem simplification necessary at this stage to keep the parameter space of our predictions to a level that allows broad applicability. The range of particle diameters contributing significantly to each IOP is identifiable from the point at which the cumulative distribution reaches an asymptotic value. In some of the modelled cases the cumulative distribution does not reach either the upper or lower asymptote (Fig. 4), indicating that the significant size range extends beyond the range of particle diameters simulated here. The impact of *n*_*i*_ on the range of particle sizes contributing to scattering (top row) and backscattering (middle row) was limited for the three PSD slopes considered here. However, the range of particle sizes contributing to absorption was strongly dependent on *n*_*i*_ and extends well beyond 10^3^ μm (1 mm) in all but one of the cases simulated here (Fig. 4, bottom row).

Generally, the significant size range affecting absorption is greater than for both scattering and backscattering, and in a number of cases potentially extends well beyond the mm size class (Fig. 4, bottom row). More importantly, we demonstrate that there are plausible circumstances where large, but numerically sparse colored particles can influence bulk IOPs, particularly absorption, and therefore affect remote sensing reflectance signals. It appears that there is no single value of maximum particle size affecting IOPs in general, or even for a specific IOP. The value varies with size distribution, refractive index and IOP. For answering our second question, why we fail to resolve the impact of large particles in IOPs, we consider maximum contributing particle diameters of 150 µm for backscattering, 2000 µm for scattering and > 10,000 µm for absorption. It needs to be emphasised that these are crude approximations only, however, these values are consistent with the previous results of Davies et al*.*^30^, considering *n*_*i*_ values used in this study.

### Why do we miss large particles with optical sensors?

**Figure 5:**
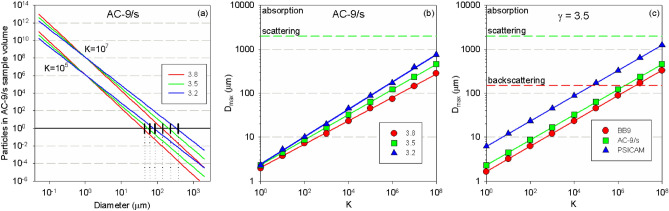
(**a**) Scaling PSD by sensor sample volume enables identification of the maximum particle diameter (D_max_) for which there is likely to be at least one particle in the sample volume. D_max_ varies with both PSD slope and concentration scaling. (**b**) Lower PSD slope values increase D_max_ for a given sensor volume. In all cases modelled here, the AC-9/s sensor does not sample the full range of particles needed to completely measure either scattering (2000 µm) or absorption (> 10,000 µm). (**c**) Sensors with different sample volumes will sample different parts of the size distribution. Larger sensor volumes (PSICAM > AC-9/s > BB9) result in higher D_max_ values for a given size distribution. The BB9 captures all sizes potentially relevant for backscattering measurements (150 µm) in only two of the cases shown here. In all other cases the optical sensor volume is too small to adequately capture all of the particles that contribute to the total scattering or absorption, with absorption being significantly more under-sampled than the other two IOPs.

In situ optical sensors typically have sample volumes of the order of ml. The WETLabs BB9 has an effective sample volume of ~ 4 ml, the WETLabs AC-9/s (25 cm pathlength) has an illuminated sample volume of ~ 12.5 ml, while a benchtop sensor such as the PSICAM^37^ has a sample volume of 400 ml. These sample volumes are too limiting to correctly capture the effect of mm-sized particles with relatively low in situ number densities. Carder and Costello first pointed out that small sample volumes and the skewed natural PSDs will mean that IOP measurements are biased towards small particles but did not provide a theoretical framework to quantify the effect^38^. Simply multiplying the PSD by the relevant sample volume provides the number density of each size class within a particular instrument’s sample volume—effectively the probability of finding a particle of that size within the sample volume. Figure 5a shows the effect of scaling several example PSDs for the sample volume of the AC-9/s. For each combination of size distribution and sensor, there is a maximum particle diameter, *D*_*max*_, at which the concentration of particles within the sample volume drops below unity. *D*_*max*_ is the largest particle size that the sensor is able to sample properly for that PSD, with larger particle sizes being systematically under-sampled due to the restricted sample volume. Low values of the PSD slope return higher *D*_*max*_ values for a given sensor volume (Fig. 5b), while larger sample volumes provide higher *D*_*max*_ values for a given PSD slope (Fig. 5c). Similarly, higher concentrations (higher *K* values) return higher values of *D*_*max*_. In all cases considered here, which cover a very broad set of conditions, *D*_*max*_ for these sensors is generally < 1000 µm (1 mm) meaning that these sensors are incapable of adequately sampling the larger size classes that have been shown above (Fig. 4) to be potentially significant contributors to bulk IOPs. For reference, a recent study^42^ found PSDs for UK coastal waters that are broadly consistent with K = 10^5^. In that case both the AC-9/s and the PSICAM would have significantly under-sampled the size ranges required for scattering and absorption, while it is likely that there may have been a less conspicuous under-sampling of particles required for backscattering by the BB9.

## Discussion

Ocean colour remote sensing reflectance signals are primarily a function of the bulk inherent optical properties of seawater and the materials found dissolved and suspended within the water column. Using Mie theory, we have clearly identified the physical basis through which large, coloured particles can affect bulk IOPs, particularly absorption, and therefore impact on remote sensing reflectance signals. The potential impact of one species of zooplankton on ocean colour signals has previously been discussed^23^. The results presented here generalise the situation to include the whole variety of mm-class colored particles found in the ocean, including other species of copepods, krill, fish eggs, fish larvae, and non-living suspended particulate material.

Here we have assumed power law PSDs of spherical particles with uniform refractive index distributions. Natural particle populations will present much more variation in refractive index, particle shape and structure, and PSD. For example, swarming and mass spawning events may breach the power law PSD, leading to even greater significance for these particles. This level of natural complexity means it is not possible to define a single size range for optical significance, but our approach does allow us to clearly state that mm-class particles are likely to make a significant contribution to bulk IOPs, particularly absorption, in many naturally occurring situations.

We have also demonstrated that standard in situ sampling approaches are biased towards smaller, more abundant particles and therefore tend to under report the impact of large particles. Large-scale or right hand truncation is often overlooked but is relatively common when sampling from power law distributions^39^, and can negatively affect associated population estimates. It is not immediately obvious how sample averaging may overcome this systematic bias. In situ sensors are typically used in a depth-profiling mode, thereby limiting time available to perform multiple measurements at any given depth. It would, of course, be possible to extend the time held at any specific depth to increase the effective volume sampled. However, the passage of a large particle through the sample volume of an AC-9/s causes a spike in readings that is generally not sampled equally at all wavebands. Similarly, passing large particles generate spikes in specific BB9 outputs. Here the issue is that each channel of the sensor observes an independent sample volume and, again, there is no consistent sampling of these occasional large particles. Interestingly, the spikes caused by such under-sampling have already been utilised to estimate presence of large particles^40,41^, but the impact of such spikes on the actual measurement of IOPs has yet to be addressed.

The benchtop PSICAM instrument is typically used to measure samples in triplicate. Extending the effective sample volume by increasing the number of samples processed is prohibitively time consuming. There are also potential artefacts associated with some species groups, e.g. zooplankton and krill, actively evading both in-flow and net sampling systems^42,43^. Current sampling procedures are clearly inadequate for resolving the influence of sparse large particles in a quantitative manner. This measurement bias potentially explains why the ocean optics community has so far overlooked the impact of large particles on ocean colour signals. The tendency of in situ observations to favour the impact of small, highly abundant particles would likely have the effect of providing confirmation bias that the smaller size range was dominant and naturally inhibit systematic investigation of the effect of larger particle sizes. However, the fact that a satellite sensor effectively samples massive volumes per pixel (100s of m^2^ multiplied by the optical depth) means that there is likely to be a mismatch between in situ and satellite observations where there are significant numbers of large, coloured particles.

New approaches to in situ IOP sensing must be developed to overcome the restricted sample volume issue. In the absence of such technology, there is a clear need to focus effort on measuring size and refractive index distributions over much broader size ranges than have previously been attempted. Technology to measure PSDs already partially exists in the form of laser diffraction^32^, flow cytometers^34^, flow cytobot^44^, flow cam^7^, holographic cameras^45^, other underwater imaging^31,46^, with combinations of these required to cover the full range of relevant sizes^1,9^. There are promising techniques emerging to determine real refractive indices from flow cytometry^34^ and microscopic imaging^47,48^. There is also scope to establish imaginary refractive indices for large particles such as *Calanus* spp. and krill using the PSICAM to measure their absorption^23^ and adapting existing inversion approaches^49^. It has recently been demonstrated that is is possible to relate measured PSDs and refractive index distributions to measured bulk IOPs^50^, so there are good grounds for pursuing this approach to ultimately link single particle measurements to ocean color satellite observations.

The results presented here demonstrate a need to renew our appreciation of the range of materials that have the potential to control ocean colour signals, and thus the interpretation of satellite ocean color observations. A requirement to carefully consider the extent to which measurements of in situ IOPs and particle size distributions fully capture the contribution of larger particles is also strikingly evident. Taken in combination, these results present an emerging opportunity to explore new applications of ocean color remote sensing in, for example, monitoring swarming (e.g. well-known krill swarms in the Southern Ocean) and spawning events (e.g. massive larval releases in major fishing grounds). The results also justify further development of in situ technology to enable full identification of all materials contributing to the optical properties of the ocean water.

We have shown that fundamental limitations in the measurement of bulk optical properties have contributed to the overlooked role of large particles in determining those signals. It is not currently possible to fully determine the relative optical significance of large particles as there are a number of contributing factors that will vary from sample to sample, some of which are not routinely available as practical measurements. However, the results presented here provide a new perspective necessary to support resolution of the dichotomy between experimental data and the evidence of visual observation of ocean color, and opens the door to a number of new avenues of research.

## Methods

### Observational data presented in Fig. 1

Figure 1c was obtained by microscope during laboratory studies of Cod egg development at SINTEF SeaLab (Trondheim, Norway), and provided courtesy of Bjørn Henrik Hansen (SINTEF Ocean).

The particle size distribution in Fig. 1d was obtained from both the LISST-100x^32^ and the SINTEF SilCam^31^ from a profiling frame lowered at 25 cm/s vertically through the water column.

Figure 1e contains an enhanced RGB image produced from MODIS data supplied by the Ocean Biology Processing Group at NASA GSFC. Real-time support from NEODAAS (NERC Earth Observation Data Acquisition and Analysis Service) enabled (cloud-free) MODIS satellite overpasses over the duration of the period in which the in situ observations were made. To generate the RGB image, wavebands 547 nm, 488 nm, and 443 nm were used, with each color scaled to include 95% of all pixels. A gamma correction of 0.9 was applied to the blue channel to improve contrast.

### Mie theory modelling

Particles in the ocean are generally more complex than simple homogeneous spheres, and natural PSDs are a complex mixture of sub-populations of widely varying composition and number densities. Replicating this level of natural complexity in a modelled environment is not currently possible. Moreover, it is not required to establish the type of fundamental behaviours discussed in this paper. This requires a model that can provide predictions of the volume scattering function (to obtain ratios of backscattering and total scattering), and absorption (including the effects of complex refractive index on scattering). While there is much debate over the assumptions of homogeneous spheres for ocean particles^10,35,51–53^, Mie theory is a reasonable first approximation for estimating bulk optical properties of natural particles^2,34,50,54^ and power-law (Junge) PSDs are similarly reasonable as a first approximation^2,3,7,33,53^.

The inputs for Mie calculations are particle diameter, *D*, and the complex refractive index, $m = n_{r} + in_{i}$ where the real part, $n_{r}$, represents the relative phase velocity of light, and the imaginary part $n_{i}$, represents extinction of light due to absorption. Following the implementation of Bohren and Huffman^55^ produces attenuation (extinction) and scattering efficiencies, *Q*_*ext*_ and *Q*_*sca*_ respectively. The absorption efficiency is then derived from *Q*_*abs*_ = *Q*_*ext*_ − *Q*_*sca*_. Mie calculations also produce values for the scattering phase function, $\tilde{\beta }\left( \theta \right)$, which is a normalized volume scattering function, such that the integral over all angles is 1. The backscattering efficiency, *Q*_*bb*_, is obtained from: $Q_{bb} = Q_{sca} \mathop \smallint \limits_{\pi /2}^{\pi } \tilde{\beta }\left( \theta \right)sin\theta d\theta$ where the term inside the integral represents the fraction of light scattered in backwards directions greater than 90 degrees. Bulk IOPs for the entire particle assemblage are calculated using:

$$ i\left( m \right) = \mathop \smallint \limits_{0}^{\infty } \frac{{\pi D^{2} }}{4}Q_{i} \left( {D,m} \right)N\left( D \right)dD $$

where *i* is one of the set (*a, b, c, b*_*b*_) representing absorption, scattering, attenuation and backscattering respectively^2^.

Calculations were performed using an incident wavelength of 670 nm (in air), a real refractive index of 1.09 (in water), 150 log-spaced diameters from 0.001 to 5000 µm, and an angular resolution of 0.5°.
